# Loss of AMPKalpha1 Triggers Centrosome Amplification via PLK4 Upregulation in Mouse Embryonic Fibroblasts

**DOI:** 10.3390/ijms21082772

**Published:** 2020-04-16

**Authors:** Qiang Zhao, Kathleen A Coughlan, Ming-Hui Zou, Ping Song

**Affiliations:** 1 Center for Molecular and Translational Medicine, Georgia State University, Atlanta, GA 30302, USA; qzhao@gsu.edu (Q.Z.); mzou@gsu.edu (M.-H.Z.); 2 Department of Science, Redlands Community College, El Reno, OK 73036, USA; kathleen.coughlan@redlandscc.edu

**Keywords:** AMPKα1, PLK4, p52, centrosome amplification, chromosomal integrity

## Abstract

Recent evidence indicates that activation of adenosine monophosphate-activated protein kinase (AMPK), a highly conserved sensor and modulator of cellular energy and redox, regulates cell mitosis. However, the underlying molecular mechanisms for AMPKα subunit regulation of chromosome segregation remain poorly understood. This study aimed to ascertain if AMPKα1 deletion contributes to chromosome missegregation by elevating Polo-like kinase 4 (PLK4) expression. Centrosome proteins and aneuploidy were monitored in cultured mouse embryonic fibroblasts (MEFs) isolated from wild type (WT, C57BL/6J) or AMPKα1 homozygous deficient (AMPKα1^−/−^) mice by Western blotting and metaphase chromosome spread. Deletion of AMPKα1, the predominant AMPKα isoform in immortalized MEFs, led to centrosome amplification and chromosome missegregation, as well as the consequent aneuploidy (34–66%) and micronucleus. Furthermore, AMPKα1 null cells exhibited a significant induction of PLK4. Knockdown of nuclear factor kappa B2/p52 ameliorated the PLK4 elevation in AMPKα1-deleted MEFs. Finally, PLK4 inhibition by Centrinone reversed centrosome amplification of AMPKα1-deleted MEFs. Taken together, our results suggest that AMPKα1 plays a fundamental role in the maintenance of chromosomal integrity through the control of p52-mediated transcription of PLK4, a trigger of centriole biogenesis.

## 1. Introduction

The accurate segregation of chromosomes during mitosis is required to correctly transmit genetic information to daughter cells. Proper chromosome segregation during mitosis is facilitated by the attachment of mitotic spindle microtubules to the centrosome, the microtubule-organizing organelle of eukaryotic cells [1], and kinetochore, which is assembled on the chromosomal centromere [2]. Abnormal centrosome number is associated with several human diseases, including primary microcephaly [3], renal cystogenesis [4], and cancer [5,6]. Centrosome amplification, with >2 centrosomes per cell, sufficiently drives chromosomal missegregation, leading to aneuploidy. This abnormal number of chromosomes in the cell [7] is associated with chromosomal instability (CIN, gain or loss of whole chromosomes) and consequent genomic destabilization [8,9]. However, the underlying mechanisms controlling centrosome over-duplication remain weakly understood.

It was reported that the Polo-like kinases (PLKs, PLK1-5) family [10] and Aurora A/B/C [11] were involved in centrosome biology and cell cycle. Among them, Polo-like kinase 4 (PLK4) is a unique member of the polo family of serine/threonine protein kinases, which is localized to centrioles and plays a well-characterized role in controlling centriole duplication [12,13] and centriole size [14] during the cell cycle. Overexpression of PLK4 results in centrosome amplification with excess centrosomes [15,16] and accelerates tumorigenesis in breast and intestinal cancer mouse models [17,18]. However, knockdown or inhibition of PLK4 results in the loss of centrosomes [19]. PLK4 levels peak only transiently during mitosis and are maintained at deficient levels during interphase [20]. PLK4 can be transcriptionally regulated by transcription factor E2F1 [21] or nuclear factor kappa B2 (NF-κB2)-p52 [22]. Kruppel-like factor 14 (KLF14) transcriptionally suppresses PLK4 expression [23]. Additionally, PLK4 is post-translationally controlled. For example, Skp-Cullin-F box (SCF) E3 ubiquitin ligase F-box protein Slimb (SCFSlimb) ubiquitinates and targets PLK4 for proteasomal degradation in *Drosophila melanogaster* [20,24]. Protein Phosphatase 2A regulatory subunit Twins (PP2ATwins) counteracts PLK4 autophosphorylation, thus stabilizing the mitotic PLK4 [25]. 

The well-known and conserved energy [26] and redox [27] sensor and regulator, adenosine monophosphate-activated protein kinase (AMPK), also exerts its critical role in cell mitosis [28,29,30]. A previous study demonstrated that AMPK deletion caused defective mitotic cells with lagging or polyploid chromosomes in a *Drosophila* model system [31], suggesting that AMPK plays a potential role in chromosomal stability. AMPK was demonstrated to localize on centrosomes [32,33]. Emerging data indicate that AMPK plays a vital role in tumor suppression [34,35,36,37,38,39]. However, the precise role of the AMPKα subunit and the mechanism by which AMPK might control accurate chromosome segregation during mitosis remain elusive. In the current study, we addressed the involvement of a major AMPKα isoform in fibroblasts, AMPKα1, in chromosome segregation by analyzing centrosome amplification in AMPKα1^−/−^ mouse embryo fibroblasts (MEFs). We present here, for the first time, that AMPKα1^−/−^ MEFs exhibit aneuploidy resulting from centrosome amplification due to p52-mediated PLK4 elevation. These findings identify an unrecognized role for AMPKα1 in chromosome integrity, providing novel insights into the mechanism of tumor suppression mediated by AMPK.

## 2. Results

### 2.1. Loss of AMPKα1 Causes Chromosome Instability and Abnormal Nuclei in Mouse Embryonic Fibroblasts (MEFs)

**Figure 1:**
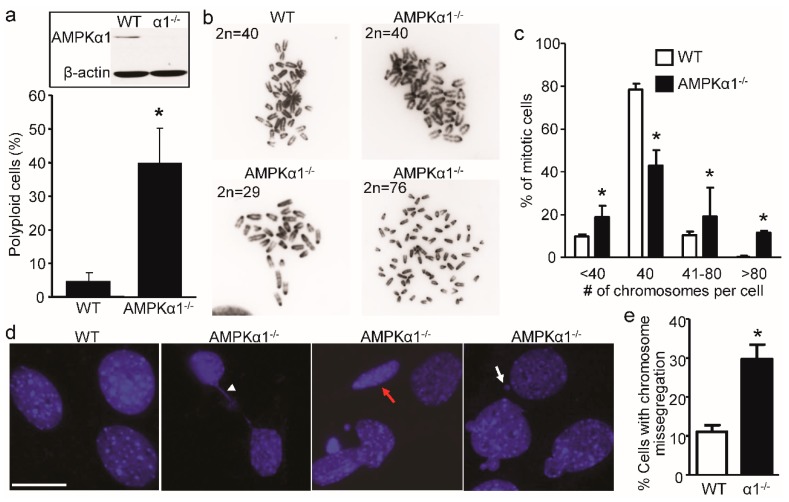
AMPKα1 deletion leads to chromosome instability in Mouse Embryonic Fibroblasts (MEFs). (**a**) Flow cytometry analysis of DNA content showed an average of about 40% polyploidy in AMPKα1^−/−^ MEFs. Data represent the mean ± S.D. from three separate experiments. * *p* < 0.01, AMPKα1^−/−^ vs. wild type (WT). Representative Western blot of AMPKα1 was shown as an inset of bar graph; (**b**) Chromosome instability in AMPKα1^−/−^ MEFs. Representative images of DAPI staining of metaphase chromosome spreads from WT and AMPKα1^−/−^ MEFs are shown; (**c**) The percentage of MEFs with different chromosome numbers per WT or AMPKα1^−/−^ MEF cell. Data represent the mean ± S.D. from four separate experiments. * *p* < 0.01, AMPKα1^−/−^ vs. WT; (**d**) The abnormal nucleus in AMPKα1^−/−^ MEFs. A lagging chromosome (white arrowhead), a flat oval nucleus (red arrow), and a micronucleus (white arrow) are shown (scale bar = 20 μm); (**e**) The bar graph shows the percentages of MEFs with chromosome missegregation. More than 100 cells were counted per group. * *p* < 0.01, α1^−/−^ vs. WT.

Given the role of AMPKα in mitosis and DNA damage [28,30,40], we analyzed whether AMPKα1 loss influences chromosome stability. First, we evaluated the ploidy status of proliferative MEFs. As depicted in Figure 1a, AMPKα1 deletion exhibited a higher frequency of aneuploidy (40% of cells) compared to WT cells regardless of the cell cycle stage. To further assess the chromosome instability induced by AMPKα1 deletion, we analyzed chromosome counts from metaphase chromosome spread. As shown in Figure 1b,c, a majority of WT MEFs had a 2n = 40, while AMPKα1^−/−^ MEFs had a wide range and highly variable number of chromosomes, ranging from 32 to 95 chromosomes. Importantly, most cells had between 38 and 50 chromosomes, indicating the loss or gain of a few chromosomes. These results indicate a possible defect in chromosome segregation, leading to chromosome instability in AMPKα1^−/−^ MEFs. 

Next, we tested if AMPKα1 affects nuclear structure. As shown in Figure 1d, AMPKα1-deleted MEFs contained various defective mitotic cells with lagging chromosomes (Figure 1d, arrowhead), a flat oval nucleus (Figure 1d, red arrow), and a micronucleus (Figure 1d, white arrow). AMPKα1 deletion in MEFs had about a three-fold increase in chromosome missegregation compared to WT (Figure 1e). These data imply that AMPKα1 is required for proper chromosome segregation and normal nuclear structure.

### 2.2. AMPKα1 Deletion Confers Centrosome Amplification

**Figure 2:**
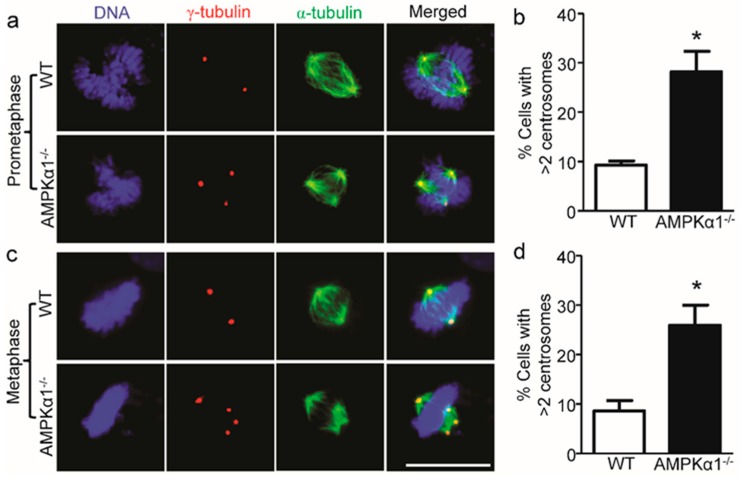
AMPKα1 deletion confers centrosome amplification in MEFs. (**a**) Representative images of centrosome morphologies of WT and AMPKα1^−/−^ MEFs (scale bar = 20 μm) in the prometaphase of the cell cycle are shown. Centrosomes, spindles, and DNA were co-stained with anti-γ-tubulin antibody (red), anti-α-tubulin antibody (green), and 4.6-diamidino-2-phenylindole (DAPI, blue), respectively, and visualized by fluorescence microscope; (**b**) Quantification data for the percentage of MEFs containing >2 centrosomes are presented. *n* = 5, * *p* < 0.01, AMPKα1^−/−^ vs. WT; (**c**) Representative images of centrosome morphologies of WT and AMPKα1^−/−^ MEFs (scale bar = 20 μm) in the metaphase of the cell cycle are shown; (**d**) Percentage of cells with >2 centrosomes. n = 5, * *p* < 0.01, AMPKα1^−/−^ vs. WT.

Centrosome amplification usually contributes to chromosome instability [8]. Next, we investigated centrosome integrity in AMPKα1^−/−^ MEFs. As shown in Figure 2a–d, AMPKα1 deletion dramatically increased the incidence of centrosome amplification (>2 centrosomes per cell) and spindle multipolarity (green, anti-α-tubulin antibody staining) in both prometaphase (Figure 2a,b) and metaphase (Figure 2c,d) of the cell cycle, while WT MEFs had regular centrosome numbers (=2) in the two phases of the cell cycle.

### 2.3. PLK4 Elevates in AMPKα1-Deleted MEFs 

**Figure 3:**
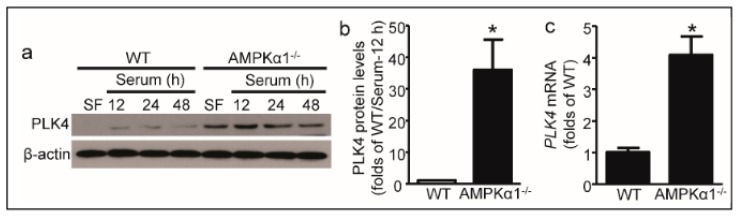
AMPKα1 deletion upregulates PLK4. (**a**) PLK4 protein levels are elevated in AMPKα1^−/−^ MEFs. SF, serum-free; (**b**) Quantification data of PLK4 protein levels in WT and AMPKα1^−/−^ MEFs. *n* = 4, * *p* < 0.01, AMPKα1^−/−^ vs. WT; (**c**) PLK4 mRNA elevated in AMPKα1^−/−^ MEFs. *n* = 3, * *p* < 0.01, AMPKα1^−/−^ vs. WT.

It is well known that PLK4 plays a critical role in centrosome integrity; hence, we inquired whether AMPKα1 regulates PLK4 expression. As shown in Figure 3a, PLK4 protein levels were markedly increased in AMPKα1^−/−^ MEFs compared with WT MEFs (Figure 3a,b). Moreover, PLK4 mRNA levels were significantly upregulated in AMPKα1^−/−^ MEFs compared with WT MEFs (Figure 3c).

### 2.4. PLK4 Upregulation in AMPKα1^−/−^ MEFs Is p52-Mediated

**Figure 4:**
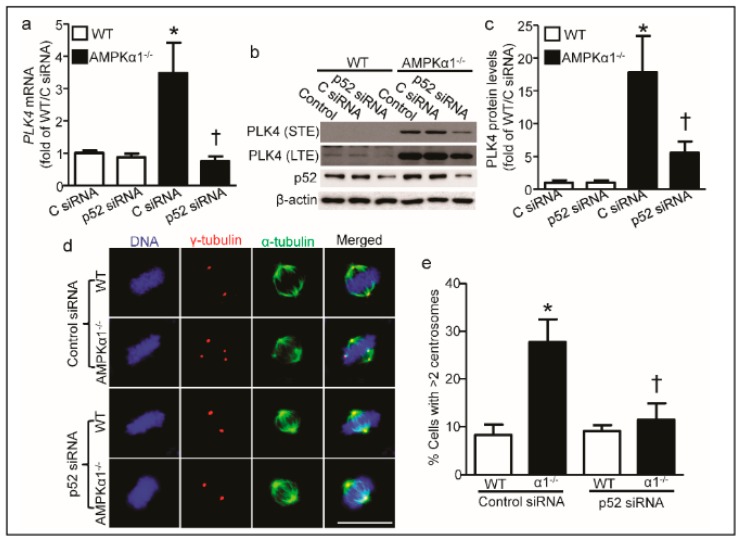
PLK4 elevation is p52-mediated. (**a**) p52 knockdown blocked the PLK4 mRNA upregulation in AMPKα1^−/−^ MEFs. *n* = 3, * *p* < 0.01, vs. C siRNA/WT; † *p* < 0.01, vs. C siRNA/AMPKα1^−/−^; (**b**) Representative Western blotting showed p52 siRNA decreased the PLK4 protein elevated in AMPKα1^−/−^ MEFs. STE: short-term exposure; LTE: long-term exposure; (**c**) Quantification data of PLK4 protein levels for (b); *n* = 4, * *p* < 0.01, vs. C siRNA/WT; † *p* < 0.01, vs. C siRNA/AMPKα1^−/−^; (**d**) MEFs were treated with p52 siRNA or control siRNA for 72 h before the start of the experiment. Cells were treated with 20 ng/mL nocodazole for 4 hours to depolymerize microtubules. Centrosomes, spindles, and DNA were co-stained with anti-γ-tubulin antibody (red), anti-α-tubulin antibody (green), and 4.6-diamidino-2-phenylindole (DAPI, blue), respectively, and visualized by fluorescence microscope (scale bar = 20 μm). Representative images are shown; (**e**) Quantification data represent the mean ± S.D. from three separate experiments. * *p* < 0.01, vs. WT/control siRNA; † *p* < 0.01, vs. AMPKα1^−/−^/control siRNA. C siRNA: control siRNA.

Next, we wanted to know the underlying mechanism for PLK4 elevation in AMPKα1^−/−^ MEFs. Since it was reported that AMPKα1 deletion upregulates nuclear factor-kappa B2/p52 [35], we tested if p52 mediates PLK4 upregulation in AMPKα1^−/−^ MEFs. As depicted in Figure 4a, p52 knockdown by specific siRNA dramatically blocked the AMPKα1^−/−^-upregulated PLK4 mRNA. Furthermore, p52 knockdown profoundly decreased PLK4 protein levels in AMPKα1^−/−^ MEFs compared with control siRNA (Figure 4b,c). Finally, p52 knockdown normalized the elevated centrosome number (Figure 4d) and decreased the percentage of cells with supernumerary centrosomes in AMPKα1-deleted MEFs (Figure 4e).

### 2.5. PLK4 Inhibition Ameliorates Centrosome Amplification in AMPKα1^−/−^ MEFs

**Figure 5:**
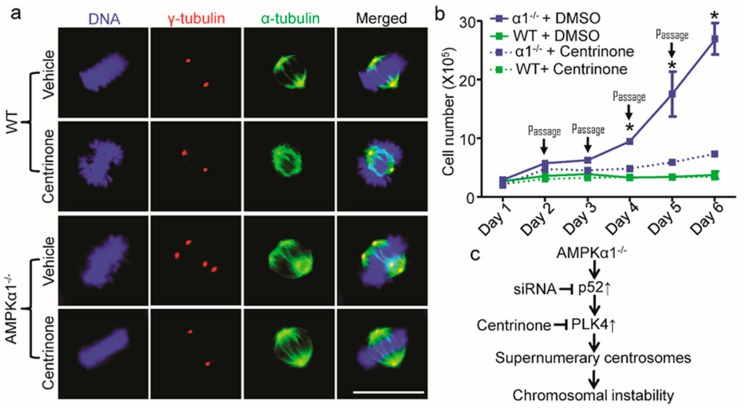
PLK4 inhibition reverses centrosome amplification and cell hyperproliferation in AMPKα1-deleted MEFs. (**a**) PLK4 inhibition by Centrinone blocked centrosome amplification in AMPKα1^−/−^ MEFs. MEFs were treated with Centrinone (125 nM) or vehicle (DMSO) for 16 h. The cells were treated with nocodazole (20 ng/mL) for 4 hours to depolymerize the microtubules. Then, centrosomes, spindles, and DNA were co-stained with anti-γ-tubulin antibody (red), anti-α-tubulin antibody (green), and 4.6-diamidino-2-phenylindole (DAPI, blue), respectively, and visualized by fluorescence microscope (scale bar = 20 μm); (**b**) PLK4 inhibition restrained cellular hyperproliferation in AMPKα1^−/−^ MEFs. MEF cells were seeded into 10-cm tissue culture dishes at 1 × 10^5^ cells/dish. Centrinone was added at 125 nM. Every day, cells were harvested, counted using a Bio-Rad cell counter, and re-seeded into new dishes. Quantification data represent the mean ± S.D. from three separate experiments. * *p* < 0.01, vs. α1^−/−^ + Centrinone; (**c**) A scheme illustrating the role of p52-PLK4 in AMPKα1 deletion-mediated centrosome amplification. P52 transcriptionally regulates PLK4, which contributes to centrosome amplification and the consequent chromosome instability.

Next, we investigated whether PLK4 mediates abnormal centrosome amplification in AMPKα1^−/−^ MEFs. As shown in Figure 5a, Centrinone, a potent and specific small molecular PLK4 inhibitor [19], effectively normalized the elevation of centrosome number in AMPKα1^−/−^ MEFs, but it had no effect on centrosome number in WT MEFs. Furthermore, Centrinone treatment markedly inhibited the hyper-proliferation of AMPKα1^−/−^ MEFs, whereas it did not alter WT MEFs proliferation performed with cellular passaging assay (Figure 5b).

## 3. Discussion

In the present study, we demonstrated that AMPKα1 deletion mediates aneuploidy in MEFs. The mechanism underlying this process is due to the elevated p52-mediated PLK4 upregulation (Figure 5c). These findings suggest that AMPKα1 is a pivotal regulator for the high fidelity of chromosome segregation.

Accumulating evidence indicates that AMPK controls the cell cycle [41,42] and mitosis [28,30] via distinct mechanisms. AMPK may control the fidelity of chromosome segregation. For example, the constitutive expression of AMPK-related kinase, NUAK1, leads to gross aneuploidies and the consequent cellular senescence in human fibroblast, WI-38 [43]. Knockdown of both AMPKα1 and AMPKα2 decreases the number and length of astral microtubules per spindle pole in human cancer cells, H1299 and HeLa [30]. Banko et al. reported that AMPKα2 phosphorylates protein phosphatase 1 regulatory subunit 12C (PPP1R12C) and p21-activated protein kinase (PAK2), which mediates the serine 19 phosphorylation of myosin regulatory light chain (pMRLC-S19) [28]. Thus, AMPK regulates actin cortex-astral microtubule attachments via pMRLC-S19 to control proper mitotic spindle orientation, which mediates accurate chromosome segregation [30]. In addition, the effect of AMPKα1 deletion on ploidy and mitotic defects were investigated in the context of liver regeneration. In contrast to the current results in MEFs, no difference in ploidy or mitotic defects (e.g., chromosomal misalignments) were observed in the AMPKα1^-/-^ regenerative liver tissue section [44]. Of note, an interesting difference between MEFs and hepatocytes is the high expression of the AMPKα2 catalytic subunit in the liver but not in MEFs [44], which may support functional redundancy and explain these discrepancies.

Although PLK1 inhibitor GW843682X blocks AMPKα activation during the cell cycle [33], it is unknown whether AMPKα is inactivated by PLK1 inhibition directly or due to the blockage of cell cytokinesis, because cell mitosis consumes energy and activates AMPK [40,45]. Here, we present that AMPKα1 deletion up-regulates the mRNA and protein levels of PLK4 dramatically, which plays a critical regulatory role in the duplication of centrioles and is involved in the correct segregation of sister chromatids [46] and standard nuclear structure [16]. Thus, PLK4 induction and defective centrosome in AMPKα1-deleted MEFs may be responsible for the lagging chromosomes and chromatin bridges [46], which might work as the primary cause of micronuclei (MN) formation and the subsequent aneuploidy [16,47]. Although p52 contributes to PLK4 upregulation, the downregulated p21 in AMPKα1-deleted MEFs [40] may also mediate the PLK4 elevation [48]. It is noteworthy that the induction of PLK4 mRNA level in AMPKα1-deleted MEFs was almost entirely restored by p52 siRNA (Figure 4a), while the PLK4 protein level in AMPKα1-deleted MEFs was partially reversed by p52 siRNA (Figure 4c), indicating that post-translational regulation may be involved in PLK4 protein induction in AMPKα1-deleted MEFs. Thus, it is warranted the investigation of the effect of AMPKα1 on PLK4 protein stability and the underlying mechanisms. 

Extra centrosomes in AMPKα1-deleted MEFs would result in aneuploidy and chromosome segregation errors that contribute to the increased DNA damage [40,49]. Incorrect chromosome separation would lead to cellular apoptosis or hyperproliferation. p52 knockdown by specific shRNA inhibits the enhanced colony formation of AMPKα1-deleted MEFs [35], suggesting that p52 upregulation plays a critical role in the hyperproliferation of AMPKα1-deleted MEFs. Additionally, AMPKα1 deletion-mediated chromosome missegregation-caused cellular hyperproliferation may be due to p21 reduction, which is consistent with p21 knockout and contributes to the growth of PLK4-overexpressing cells [50].

Loss of AMPKα1 culminates in centrosome amplification and spontaneous chromosomal abnormalities-deranged genome instability in fibroblasts. Recent studies from Li et al. have uncovered a previously unrecognized function of AMPK in protecting the replication fork structure upon replication stress to ensure genome stability [51]. As AMPK is an essential modulator of cell metabolism and redox balance, it will be interesting to examine whether AMPKα1 regulates centrosome integrity-associated genomic fidelity, in part via modulating cellular redox balance. Interestingly, AICAR (5-aminoimidazole-4-carboxamide ribonucleoside), a well-known AMPK activator [52], selectively induces the apoptosis of aneuploid (trisomic) MEFs and human cancer cell lines [53]. Since aneuploidy is a hallmark of cancer, AMPK may be a promising and selective drug target for aneuploidy-related cancer therapy.

In conclusion, our studies revealed an important role for AMPKα1 in cell biology and connected two hallmarks of human cancer: chromosomal instability and hyperproliferation. Given the importance of AMPK in the cell cycle, these findings hold profound implications for understanding the molecular mechanisms, by which AMPK acts as a potential tumor suppressor. 

## 4. Materials and Methods 

### 4.1. Materials and Reagents 

The following antibodies were purchased from Cell Signaling Technology (Beverly, MA, USA): rabbit anti-PLK4 (3258), rabbit anti-p52 (4882), rabbit anti-α-tubulin (2125), anti-rabbit IgG-HRP (7074), and anti-mouse IgG-HRP (7076). Mouse anti-PLK1 antibody (ab17057) was from Abcam (Cambridge, MA, USA). Mouse anti-β-actin antibody (sc-47778) was obtained from Santa Cruz Biotechnology (Santa Cruz, CA, USA). Mouse anti-γ-tubulin antibody (MA119421) was from Thermo Scientific (Rockford, IL, USA). NFκB p52 siRNA (m) (sc-36043) and control siRNA (sc-37007) were from Santa Cruz Biotechnology. Other chemicals and organic solvents of the highest available grade were obtained from Sigma-Aldrich (St. Louis, MO, USA). AMPKα1^−/−^ mice were described elsewhere [54,55]. Mice were handled following animal protocols approved by the Institutional Animal Care and Use Committee of the University of Oklahoma Health Sciences Center (Oklahoma City, OK, USA). 

### 4.2. Cell Culture and Transfection

Mouse embryonic fibroblasts (MEFs) were isolated from AMPKα1^−/−^ and WT mouse embryos at 13.5 days post-coitus, and cells were spontaneously immortalized by the 3T3 protocol, as described previously [56,57]. A 13.5-day mouse fetus was decapitated, and the liver and heart were removed. The remaining part of the fetus was washed by sterile PBS, thoroughly minced, and trypsinized (0.25% trypsin-EDTA, Cat. No. 25200-072, Thermo Fisher Scientific, Grand Island, NY, USA). The dissociated cells were resuspended. To immortalize MEFs, cells were passaged serially according to the 3T3 protocol (3 × 10^5^ cells were plated in a 60 mm tissue culture dish every three days) until the proliferation rate in the culture stabilized. Cells were then cultured for an additional 15 passages (to about passage 35) and, at that point, were considered immortalized and used for experiments. MEFs were cultured in Dulbecco’s modified Eagle medium (DMEM, Invitrogen, Carlsbad, CA, USA) supplemented with 10% *v*/*v* FBS, L-Glutamine (2 mM) (Lonza, Walkersville, MD, USA), penicillin (100 U/mL), and streptomycin (100 μg/mL) (Life Technologies, Grand Island, NY, USA). For cell synchronization in the G0/G1 phase, MEFs were starved with a serum-free medium for 48 h and then re-incubated with complete medium for the indicated times.

### 4.3. Indirect Immunofluorescence Analysis 

Cells were grown to exponential phase on poly-L-lysine-coated glass coverslips and then were fixed with 4% paraformaldehyde in PBS, permeabilized with 0.1% Triton X-100 in PBS or image-IT Fix (Cat. No. R37602, Invitrogen, Carlsbad, CA, USA), and blocked with 3% bovine serum albumin (BSA) in PBS. Primary antibodies used were rabbit anti-α-tubulin (1:100 *v*/*v*) and mouse anti-γ-tubulin (1:100 *v*/*v*). DNA was stained with antifade reagent with 4′,6-diamidino-2-phenylindole (DAPI) (Invitrogen, Carlsbad, CA, USA). For indirect immunofluorescence, Alexa Fluor® 488 or 555 were used for the detection of protein. Confocal microscopy was performed using a Zeiss 710 confocal microscope (Jena, Germany) with a 100× oil immersion lens. Image editing was performed in Adobe Systems Incorporated (San Jose, CA, USA).

### 4.4. RNA Extraction, cDNA Synthesis, and Real Time PCR

Total mRNA was isolated and purified using the RNeasy mini kit from Qiagen (Valencia, CA, USA) according to the manufacturer’s instructions. cDNA was synthesized from isolated mRNA using the iScript cDNA synthesis kit (Bio-Rad Laboratories, Hercules, CA, USA), as described previously [55] and following the manufacturer’s instructions. Real-time PCR was performed on a CFX96 Touch™ Real-Time PCR Detection System (Bio-Rad, Hercules, CA, USA) with SYBR Green PCR Master Mix (ThermoFisher Scientific, Waltham, MA, USA) and one μl of the first-strand cDNA as a template with specific primers for PLK4 (5′-GAGCGTGAATAGTGCCGCTTTC-3′, 5′-TGAACCCACACAGCTCCGCTAG-3′). The levels of gene expression were determined relative to that of GAPDH (5′-AAGGTCATCCCAGAGCTGAA-3′, 5′-CTGCTTCACCACCTTCTTGA-3′).

### 4.5. Metaphase Chromosome Spreads

MEFs growing in an exponential phase were incubated with 0.01 µg/ml colcemid (Calbiochem, Gibbstown, NJ, USA) for 3 h. Cells were harvested by trypsinization, swollen in 75 mM KCl at 37 °C for 20 min, fixed with 3:1 methanol/acetic acid (*v*/*v*), and dropped onto clean, ice-cold glass microscope slides [58]. The slides were air-dried and stained with DAPI for 10 min. Chromosome numbers were evaluated using a microscope (BX53TF; Olympus, Tokyo, Japan) and a digital camera (DP80; Olympus, Tokyo, Japan) under a 100×/1.4 oil objective. At least 50 metaphases were analyzed per sample.

### 4.6. Flow Cytometry Analysis

Exponentially growing cells were collected by trypsin digestion and fixed in ice-cold 80% ethanol in PBS for at least 2 h. Cells were stained with 50 µg/mL propidium iodide (PI) in the presence of 10 µg/mL DNase-free RNase. Cell cycle profiles were determined by FACSDiVa (Becton-Dickinson Bioscience, San Jose, CA, USA), and data were analyzed using FCS Express V3 software (Los Angeles, CA, USA). For the analysis of aneuploidy, a Multicycle plug-in was used.

### 4.7. Protein Extraction and Western Blotting

Whole-cell extracts were prepared by employing cell lysis buffer (9803) from Cell Signaling Technology with protease and phosphatase inhibitor cocktails I and II (Cat. # BP-479 and BP-480, Boston BioProducts, MA, USA). Protein samples (30–60 μg) were loaded and separated by SDS-PAGE and transferred onto nitrocellulose membranes. The membrane was blocked by 5% nonfat powdered milk in TBST (50 mM Tris, pH 7.5, 150 mM NaCl, 0.1% Tween 20) for 30 min and then probed with different antibodies in 4% powdered milk in TBST, as previously described [59]. The membrane was washed extensively with TBST, and then incubated with the appropriate horseradish peroxidase (HRP)-linked secondary antibodies (Cell Signaling Technology, Beverly, MA, USA). Signals were visualized with an enhanced chemiluminescence detection system (GE Healthcare, Pittsburgh, PA, USA) and quantified by densitometry. Equal loading of protein was verified by immunoblotting with the anti-β-actin antibody. 

### 4.8. Passaging Assays

Cellular passaging assays were performed as previously described [19]. Briefly, MEF cells were seeded into 10 cm tissue culture dishes at 1 × 10^5^ cells/dish. Centrinone was added at 125 nM. Total cells were harvested, counted using a Bio-Rad cell counter, and re-seeded daily into new dishes. 

### 4.9. Statistical Analysis

Data were presented as mean ± S.D. Differences between multiple means were evaluated by two-tailed Student’s *t*-test or analysis of variance with post hoc Bonferroni corrections. A *p* value < 0.05 was considered statistically significant. 

## 5. Conclusions

AMPKα1 maintains chromosomal integrity through the control of p52-mediated transcription of PLK4, a trigger of centriole biogenesis.

## Abbreviations

AMPK	adenosine monophosphate-activated protein kinase
CIN	chromosomal instability
MEFs	mouse embryonic fibroblasts
PLK4	Polo-like kinase 4
